# Numerical Analysis of Aggregate Debonding in Asphalt Concrete

**DOI:** 10.3390/ma18102297

**Published:** 2025-05-15

**Authors:** Marek Klimczak, Marta Oleksy

**Affiliations:** Faculty of Civil Engineering, Cracow University of Technology, 31-155 Cracow, Poland; marta.oleksy@pk.edu.pl

**Keywords:** finite element method, asphalt concrete, contact, viscoelasticity, Prony series, image processing

## Abstract

The overall response of asphalt concrete under a subjected load is governed not only by the properties of its constituents but also by the interactions among them. In this paper, we focus on the numerical analysis of aggregate debonding, which is typically a phenomenon that precedes crack initiation. The interfacial transition zone plays a crucial role in the macroscopic performance of this material. Using image processing to reconstruct a specific sample microstructure, we carried out several finite element analyses to assess the impact of the debonding phenomenon on the general performance of asphalt concrete. Image segmentation algorithms were employed to accurately detect aggregate boundaries, followed by vectorization to describe their geometries. After applying a series of error-controlled geometry simplification procedures, the final microstructure was exported to the ABAQUS/Standard 2023 environment. A linear elastic solution for the reconstructed asphalt concrete sample was used as the reference solution. It was compared with linear viscoelastic solutions with a perfect bonding between constituents and, in the next step, with debonding allowed at aggregate–matrix interfaces. The latter phenomenon was analyzed by enforcing respective contact conditions between the aggregate and the bituminous matrix. It was found that introducing the viscoelastic material model for mastic resulted in a 142.72% increase in the vertical extreme displacement relative to the purely elastic solution. When debonding effects were additionally considered, this increase rose to 188.44%. The results confirm the necessity of debonding conditions to be introduced in reliable finite element analyses of asphalt concrete.

## 1. Introduction

Recent advancements in computational resources allow for the incorporation of heterogeneities observed at increasingly smaller scales into numerical analyses. Asphalt concrete (AC), a composite material consisting of bituminous binder, aggregate, filler, and other optional additives, can nowadays be modeled with detailed insight into the processes occurring at the scale(s) lower than the primary analysis scale. In numerical modeling, “lower scale impact” is considered in a manifold manner.

### 1.1. State of the Art

#### 1.1.1. Mechanistic–Empirical Pavement Design Method

In the mechanistic–empirical pavement design method [1,2], asphalt pavement structure is typically represented as a multi-layered elastic domain, where each layer is assumed to be homogeneous and characterized by effective material properties. Asphalt pavement structure fatigue performance is evaluated based on the response to a single loading simulation. A set of empirical formulas is applied to take into account diverse effects that may influence the final bearing capacity of the whole pavement structure. Among them, some factors are associated with the lower-scale phenomena. However, as highlighted by Kim [3], this approach may be insufficient in some cases. In particular, this observation refers to the newly introduced materials and innovative layer compositions that were not covered by the previously proposed empirical equations.

#### 1.1.2. Continuum Models

Similarly to the mechanistic–empirical pavement design method, several studies and applications [4,5,6,7,8,9,10] have developed effective constitutive models to reliably describe the behavior of various asphalt mixtures. Similarly to the mechanistic–empirical design, this method consists of the consideration of the lower-scale phenomena in a smeared manner.

For example, Collop et al. [4] studied the elastic–viscoplastic response of asphalt pavement layers incorporating damage evolution based on the loading history, time-dependent degradation of binder properties, and the progressive loss of adhesion between the binder and aggregates. Generally, the overall response of the asphalt pavement layers accounts for the internal scale phenomena in an implicit way via constitutive model parameters.

In [5], a continuum damage model was developed to characterize fatigue behavior in asphalt cement and mastic. Therein, fatigue cracking was reported to be the primary mode of asphalt pavement failure. Even though the developed model is based on the assumption of domain continuity, its parameters (particularly those associated with damage evolution) are related to microstructural changes. As it is stated in [5], radial microcracking is the dominant phenomenon responsible for the progressive reduction in the stiffness of asphalt mixtures. This process is described by the damage evolution law, where the microstructural changes are accounted for in the form of the internal state variable, denoted as S.

The application of the viscoelastic continuum damage to modeling the fatigue performance of asphalt pavements was presented in [10]. As in [5], the internal state variable S was introduced to quantify the effect of the microstructural cracking, which results in stiffness degradation. This approach originates from Schapery’s nonlinear viscoelastic theory [11]. In this framework, damage characteristic curves—relating the internal state variable *S* to the pseudo secant modulus *C*—are employed to describe the material response under loading. In that study [10], satisfactory results were obtained comparing the evaluated and observed fatigue performance of the analyzed asphalt pavements.

In [6,8,9], macroscopic analyses were also conducted that incorporated microscale phenomena through the use of effective material parameters. As highlighted in [9], a commonly adopted fatigue failure criterion for asphalt pavements is the 50% reduction in the material’s initial stiffness. Referring to the microstructure, such a criterion denotes the material state when the microcracks’ propagation process ends and the macrocracks begin to propagate [9].

In [7], the asphalt mastic modulus was evaluated based on micromechanical observations. Even though the macroscopic analysis was carried out with the assumption of domain homogeneity, some specific observations from the lower scale were used to evaluate the macroscopic parameters. In particular, the physicochemical interactions between filler particles and the bitumen were explicitly considered. The background of this idea is the observation that other micromechanical-based models underestimate the performance of asphalt cement and mastic. A stiffening effect of the aforementioned physicochemical interaction, up to some filler concentration, allows one to evaluate specimen performance in a more adequate manner.

#### 1.1.3. Multiscale Modeling

From the numerical modeling point of view, the approaches discussed in the preceding sections share a common underlying principle. Explicitly accounting for the heterogeneous internal structure at lower scales within a macroscopic analysis is computationally intensive—and often prohibitive—particularly when nonlinear phenomena at those scales are involved. To address this challenge, the analyzed domain is typically assumed to be continuous and homogeneous at the macroscopic scale. This simplification allows for the use of constitutive models with effective parameters that implicitly capture the influence of microscale behavior. In this way, lower-scale phenomena are incorporated into the analysis in a smeared or averaged manner, both through the form of the constitutive model and the calibration of its parameters. This modeling strategy significantly improves computational efficiency, making numerical simulations feasible within practical time constraints.

In order to account for the local inhomogeneities without losing the numerical efficiency of the computations, various multiscale methods are used. Even though detailed descriptions of the developed methods are beyond the scope of this paper, some most important aspects are highlighted below. Additionally, the two selected methods are depicted schematically to present some possible advantages of this approach. For a comprehensive review of other methods, we refer to [12,13].

As far as the microstructure of asphalt concrete is concerned, it is a set of irregularly shaped aggregate particles that are embedded in mastic. Additionally, the particles are randomly distributed and oriented. In order not to introduce oversimplifications at the step of geometry modeling, the AC microstructure is reconstructed using image processing techniques [14,15] or virtually generated in a justified manner [16,17]. Thus, no periodicity can typically be observed in the microstructure.

Following the observations presented above, the multiscale method selected for the analysis should be free of simplifying assumptions referring typically to the shape and periodicity of the domain. Among others, two groups of methods particularly fulfill this requirement: the computational homogenization [18,19,20] and the multiscale finite element method [21,22].

The concept of computational homogenization is based on the representative volume element (RVE). An RVE is a small subdomain that encapsulates all the essential characteristics of the surrounding volume and is associated with a Gauss integration point. In a recursive manner, the macroscale deformation at this point is used to impose the RVE boundary conditions for the boundary value problem (BVP) to be solved at the microscale. Consequently, the quantities of interest are averaged over the RVE domain and transferred to the corresponding Gauss point at the macroscale. This method is well established and widely used [18], and applications for asphalt concrete can be found (see, e.g., [20]). The parallelization of computations is the cure for a possible huge set of Gauss points and corresponding RVE subproblems to be solved. A similar approach called numerical homogenization was developed in the context of asphalt concrete in [19]. The simplification of the computations in the numerical homogenization is based on the fact that effective material properties are computed once per analysis using a set of predefined numerical tests.

Another effective method for modeling heterogeneous materials with irregularly shaped and randomly distributed inclusions is the multiscale finite element method (MsFEM) [17,21,22]. Its applications to asphalt concrete numerical modeling can be found in [17,22]. The idea of this method involves two levels of discretization: actual computations are conducted on a macroscale finite element mesh, which is relatively coarse. Effective element matrices and vectors are calculated elementwise using special shape functions that incorporate the internal microstructural heterogeneity within the macroscale elements. This framework also typically employs parallelization to enhance computational efficiency.

Moreover, increasing the approximation order in both of the briefly described methods can further improve the convergence rate and reduce modeling error. Studies exploring these enhancements can be found in [17,19,22].

#### 1.1.4. Direct Finite Element Analysis

The discussion presented in the previous section refers to the situation when numerical efficiency cannot be guaranteed due to the large dimensions of the analyzed domain with irregularly shaped and randomly distributed, and the nonlinear behavior of the constituents. Numerically, such an elaborate analysis can be prohibitively expensive in the context of computational time.

However, by limiting the spatial dimensions of the specimens under study, it becomes feasible to investigate the complexities of composite behavior without resorting to significant simplifications—neither in the geometry of the microstructure nor in the material models employed. The finite element method is commonly used to solve such initial-boundary value problems within heterogeneous subdomains. Some applications to asphalt concrete modeling can be found in [3,4,10,16], among others.

**Figure 1:**
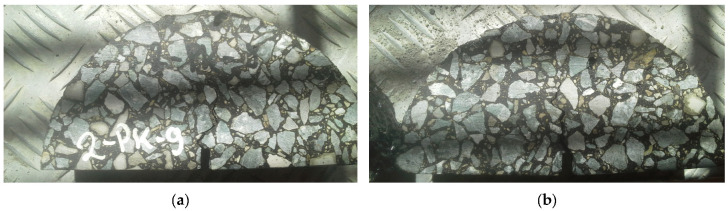
Specimens after the SCB test: (**a**) first specimen; (**b**) second specimen.

In this study, we aim to analyze the interaction between the primary constituents of asphalt concrete, specifically aggregate particles and the mastic. As reported in [23,24], the interfacial transition zone (ITZ) is often regarded as the “weakest link” in asphalt concrete composites, with a typical thickness of approximately 5 to 20 µm. Crack initiation commonly occurs at the interface between the aggregate and mastic. In Figure 1a,b, a typical failure mode in a semi-circular bending (SCB) test is presented.

In this type of test, the approximate localization of the first is predetermined by the manufactured notch. However, the crack path cannot be evaluated in advance. One can observe in Figure 1a,b that the crack initiated along the boundary of the aggregate particle close to the notch. Furthermore, the crack path is “attracted” to the interfacial transition zone between the constituents, highlighting the critical role this region plays in crack propagation.

### 1.2. Scope of the Study

In the present literature, there is a lack of studies that aim to consider most of the aspects associated with the microscale of AC with the same precision. Typically, the focus is on one selected aspect, which is exploited in detail. Consequently, some idealizations are introduced to other aspects of numerical modeling. In our research, we aim to present a consecutive development of the AC modeling oriented both on reliability and numerical efficiency.

In this study, we analyzed the impact of the aggregate debonding on the overall behavior of the AC specimen. Firstly, the microstructure was reconstructed using image processing techniques and then transferred to ABAQUS/Standard 2023 through proprietary Python 3.12 code, where finite element analyses were conducted. In the context of the previous discussion on the approaches to microstructure modeling, we did our best to reconstruct real specimens in a reliable manner. Additionally, due to the limited spatial dimensions of the analyzed specimens, we performed a direct finite element analysis accounting for the digitally reconstructed microstructure.

Secondly, we built the final numerical model in a hierarchical way, i.e., we enhanced the linear elastic model introducing the viscoelastic aggregate behavior, and ended up with the latter model further enhanced with contact modeling between the mastic and aggregate. The applied material models employed in the simulations are discussed in the next section.

## 2. Materials and Methods

### 2.1. Asphalt Concrete Specimen

In this analysis, we used high-quality images of asphalt concrete AC 16 type specimen with a typical gradation curve, without any special additives in the mixture. The specimens were prepared in accordance with [25] for further SCB test purposes.

Specifically, we used the specimens that were prepared and further underwent the SCB testing in the laboratory of the Chair of Highway, Railway, and Traffic Engineering of Civil Engineering Faculty (Cracow University of Technology, Cracow, Poland).

### 2.2. Digital Reconstruction of Asphalt Concrete Microstructure

In order to study the realistic AC microstructure, we used high-quality images (with a resolution of 24 Mpx) of the actual specimens described above. Schematically, we followed the flowchart presented in [26] and other similar research studies to reconstruct the internal structure of the specimen. For the sake of simplicity, we limited the analysis to a 2D case. However, it can be generalized to a full 3D problem with a reconstruction based on a set of X-ray computed tomography (XRCT) scans [20].

**Figure 2:**
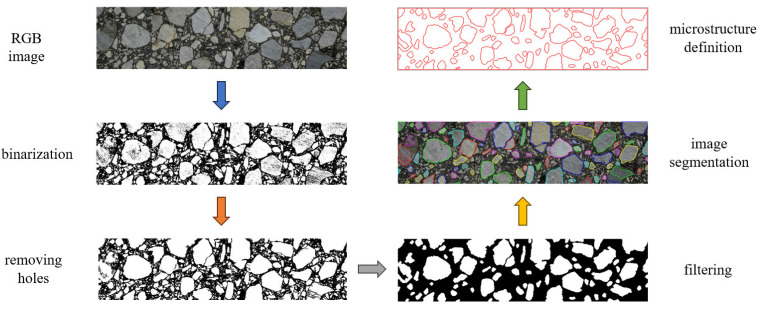
Flowchart of the digital microstructure recognition (the same specimen is used for numerical tests); the arrows indicate the direction of the processing.

After taking a high-quality photograph of the specimen, the image was processed as follows (see Figure 2):

- The RGB image was converted to a grayscale form;
- Binarization was performed to distinguish only two phases;
- A threshold was set to eliminate objects smaller than 2 mm, typical for AC;
- Unrealistic holes in the aggregate particles were removed;
- The boundaries of the aggregate particles were detected and stored in vector graphics format.

Numerical efficiency can also be significantly enhanced through error-controlled simplifications of the boundary geometries. In [27], we developed an algorithm for generating simplified aggregate boundaries based on the initially reconstructed geometry. In the error-controlled mode, a new polygon is created from the initially captured vertices. The number of the new polygon vertices is defined in such a way that the discrepancy between the areas of both objects is at an acceptable level. In this research, five iterations were used to obtain the microstructure geometry, which was then transferred to ABAQUS/Standard 2023 via a Python 3.12 script.

### 2.3. Prony Series Linear Viscoelastic Model

In continuum mechanics, viscoelasticity is a fundamental property that describes how materials exhibit both viscous and elastic characteristics when subjected to deformation. Viscous behavior is related to energy dissipation, while elastic behavior involves the material returning to its original state after deformation.

To predict the viscoelastic properties of materials, two fundamental mechanical models are commonly used: the Voigt model and the Maxwell model. These models are represented by combinations of springs (representing elastic components) and dashpots (representing viscous components). These elements can be connected in parallel or series, or in more complex combinations, to capture different material behaviors under stress or strain.

In the generalized Maxwell model (spring and dashpot connected in series), each Maxwell element contributes to the relaxation modulus in a manner characterized by the following equation:

(1) $$G_{i}\left(t\right)=G_{i}e^{-\frac{t}{\tau_{i}}},$$

where $G_{i}$ is the spring constant of the *i*-th Maxwell element and $\tau_{i}$ is the relaxation time associated with that element. The relaxation time is related to the dashpot constant $\eta_{i}$ and the spring constant by the following equation:

(2) $$\tau_{i}=\frac{\eta_{i}}{G_{i}},$$

This relationship indicates how the material’s viscoelastic response is governed by both the viscous and elastic properties of the material. When multiple Maxwell elements are connected in parallel, the overall relaxation modulus $G\left(t\right)$ of the material is the sum of the contributions from each Maxwell element, yielding the following generalized form:

(3) $$G\left(t\right)=\sum_{i=1}^{n}G_{i}e^{-\frac{t}{\tau_{i}}}\;,$$

An extension of the generalized Maxwell model involves the generalized Maxwell–Wiechert model [28], which adds an additional spring term to account for long-term elastic behavior. This model is represented by the relaxation modulus [29,30] in the following form:

(4) $$G\left(t\right)=G_{\infty }+\sum_{i=1}^{n}G_{i}e^{-\frac{t}{\tau_{i}}}\;,$$

where $G_{\infty }$ represents the equilibrium modulus of the material, reflecting the elastic behavior at very long times (when all the viscous effects have relaxed). This extended form is also known as the Prony series [31,32], a mathematical tool commonly used in viscoelasticity to describe the time-dependent stress–strain relationship.

The Prony series is especially valuable in modeling materials that exhibit both time-dependent stress relaxation and creep behavior under constant stress. The linear viscoelastic model assumes that the stress–strain relationship of a material is linear, but it is time-dependent. The Prony series, as a part of the generalized Maxwell and Maxwell–Wiechert models, provides an effective way to describe how materials respond to external forces over time.

In this research, ABAQUS/Standard 2023 is employed, incorporating the Prony series to define and model viscoelastic materials, enabling an accurate representation of their time-dependent behavior. The generalized Maxwell model, often expressed using the Prony series, can be implemented in ABAQUS/Standard 2023 as part of the material’s viscoelastic definition. This is typically performed using the elastic–viscoelastic (standard solid) model, which allows for the specification of the material’s relaxation behavior over time.

### 2.4. Contact Modeling

Contact modeling is a crucial component of computational mechanics that describes how bodies interact when they come into contact. This phenomenon plays a fundamental role in engineering disciplines, especially in structural analysis and material science.

One of the key difficulties in contact mechanics is its inherent nonlinearity, which arises due to factors like significant deformations, material properties, and frictional effects. Numerical approaches, especially those employed in finite element analysis (FEA), provide effective solutions to manage these challenges. Advanced simulation software like ABAQUS enables engineers to model contact interactions using sophisticated algorithms based on well-established contact theories, including Hertzian contact mechanics and Coulomb friction models.

When two objects come into contact, their interaction is mathematically represented through a gap function, which defines the normal distance between the surfaces. This function is given by the following:

(5) $$g_{n}=\left|x_{2}-x_{1}\right|,$$

where $x_{1}$ and $x_{2}$ denote the positions of corresponding points on the contacting surfaces. Three primary scenarios define the contact condition:

- If $g_{n}$ > 0, the surfaces are not in contact;
- If $g_{n}=$ 0, contact is established;
- If $g_{n}$ < 0, interpenetration occurs, which is physically unrealistic and must be prevented.

To enforce this constraint, a normal contact force is introduced to eliminate penetration.

Finite element methods (FEMs) provide an effective numerical framework for solving contact-related problems. Two common approaches for enforcing contact constraints are the penalty method and the Lagrange multiplier method [33].

The penalty method introduces an artificial stiffness parameter, allowing for minor penetration. Although computationally efficient, it may introduce numerical compliance, which can affect accuracy.

The Lagrange multiplier method strictly enforces the contact condition by introducing additional unknowns, ensuring that no penetration occurs. While more precise, it increases computational cost due to the extra constraint equations [34].

However, in practical applications, contact interactions are often accompanied by friction, which resists relative tangential movement between surfaces.

A frictional contact formulation is introduced, e.g., in [35], based on an approximation of the tangent matrix.

Modern finite element software, such as ABAQUS/Standard, offers the robust implementations of contact mechanics theories. The two main contact formulations in ABAQUS/Standard include the following:

- Penalty Contact (Soft Constraint): Allows slight penetration by introducing a stiffness parameter;
- Lagrange Multiplier Contact (Hard Constraint): Ensures strict non-penetration for higher accuracy at the cost of increased computational effort.

The choice of contact formulation depends on accuracy needs and computational limitations. Hard contact models offer precise results but demand more processing power, while soft contact models strike a balance between efficiency and time.

In this research, ABAQUS/Standard 2023 is employed to simulate the behavior of elastic and viscoelastic materials and to model the contact problem in asphalt at the interface between inclusions (aggregate particles) and the matrix (mastic).

## 3. Results

### 3.1. Initial Test

In the first example, a tensile test was conducted under plane strain conditions. The considered domain was a rectangle (0.5 m × 0.25 m), with symmetry conditions applied to its left and bottom boundaries (displacements disallowed in a direction perpendicular to the given edge). A constant pressure of 0.1 MPa was applied in the x-direction on the right edge, while the top edge remained free. The analysis period was set to 180 s.

Two materials were examined in this study. The first was an elastic material characterized by mastic parameters, with a Young’s modulus of 385.9574 MPa and a Poisson’s ratio of 0.2. The second material followed a viscoelastic Maxwell model, incorporating an additional viscous component defined by the Prony series, with the parameters detailed in Table 1.

**Figure 3:**
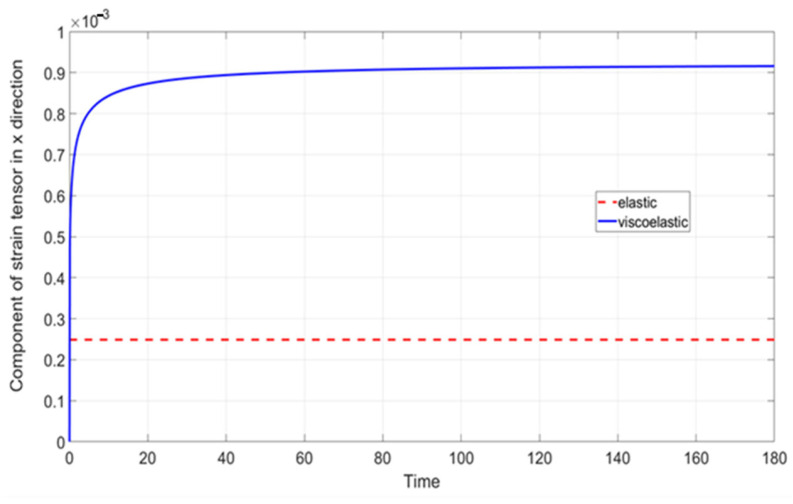
Comparison of $\varepsilon_{xx}$ strain tensor components for the adopted elastic (red dashed line) and viscoelastic (blue solid line) material model as a function of time.

The relationship between strain and time increment was analyzed and is presented in Figure 3. For the elastic material, a constant strain value was observed. In contrast, the viscoelastic material exhibited a rapid initial strain increase, followed by a gradual and asymptotic rise over time.

Figure 3 presents a typical comparison between elastic and viscoelastic materials. It highlights the limited applicability of the linear elastic material model in the context of mastic analysis, a limitation that becomes even more evident when accounting for temperature fluctuations.

### 3.2. Test 1—AC Linear Elastic Analysis

**Figure 4:**
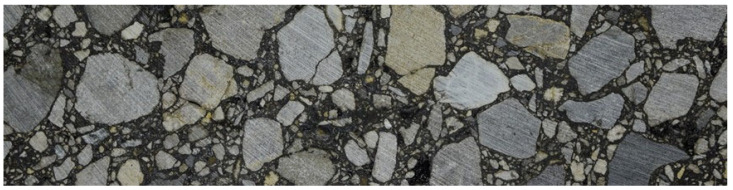
Analyzed AC specimen image.

In the second example, a heterogeneous domain containing aggregate particles (inclusions) embedded in mastic (matrix) was analyzed based on the geometry obtained from the analyzed image, as described in Section 2.2 and the corresponding references. Specifically, the image used for the specimen microstructure recognition is shown in Figure 4. Its physical dimensions are equal to 0.20 m × 0.05 m.

**Figure 5:**
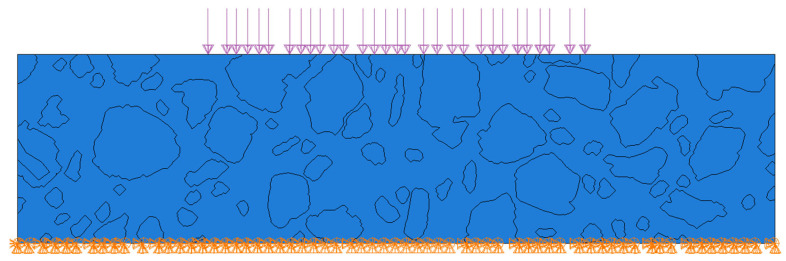
Analyzed domain with imposed boundary conditions.

For this test, both materials were assumed to be elastic. The mastic was characterized by the same Young’s modulus and Poisson’s ratio as in the initial test (Section 3.1), while the inclusions possessed properties like crystalline quartz, with a Young’s modulus of 70,000 MPa and a Poisson’s ratio of 0.17. The bottom part of the domain was fixed, while the top edge (its central part of length of 0.1 m) was subjected to a pressure load of 0.35 MPa. The considered domain is presented in Figure 5.

The finite element model includes 32,100 nodes and 29,869 plane strain elements, consisting of linear quadrilateral and linear triangular elements. This combination provides sufficient flexibility to accurately represent complex geometries while maintaining computational efficiency. The mesh was designed with particular attention to element quality and density to ensure that its influence on the simulation results is negligible, thus providing reliable outcomes for the plane strain analysis.

**Figure 6:**
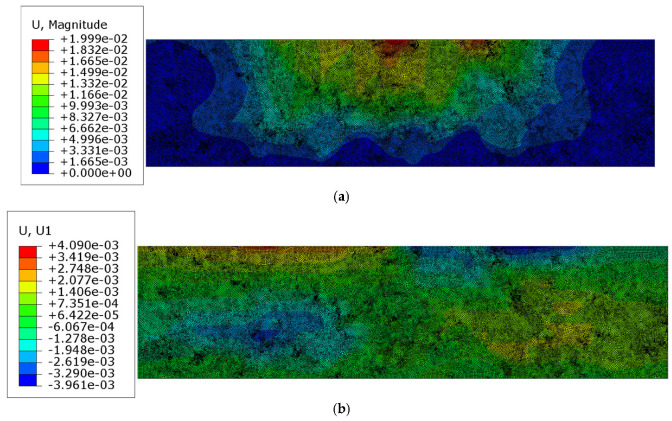

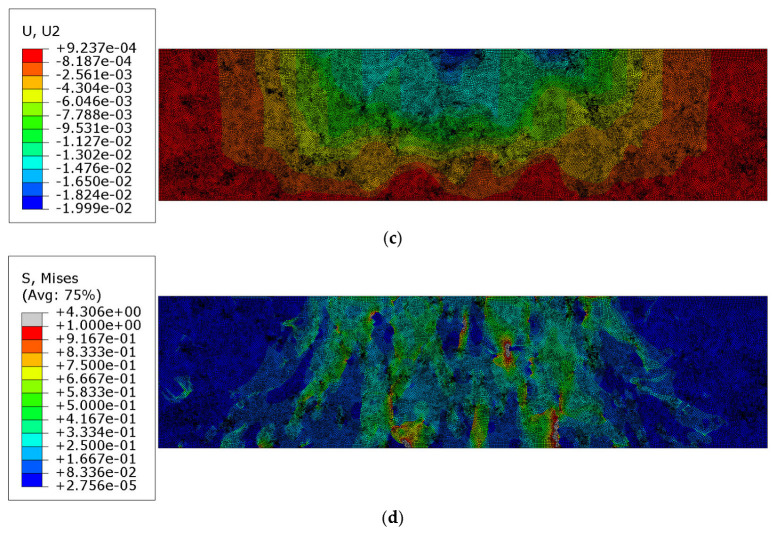
Results of Test 1: (**a**) displacement magnitude [mm]; (**b**) horizontal displacement component $u_{x}$ [mm]; (**c**) vertical displacement component $u_{y}$ [mm]; (**d**) Mises stress [MPa] in the truncated range.

In this test, the obtained displacement results and the reduced Mises stress distribution are presented below in Figure 6a–d. For clarity, the Mises stress distribution is shown only in the truncated range. 

As it was discussed in Section 3.1, the analysis of asphalt concrete behavior only in the elastic range is insufficient. Therefore, the results presented in this section serve as the reference ones for the gradually enhanced analysis.

### 3.3. Test 2—AC Linear Viscoelastic Analysis

**Figure 7:**
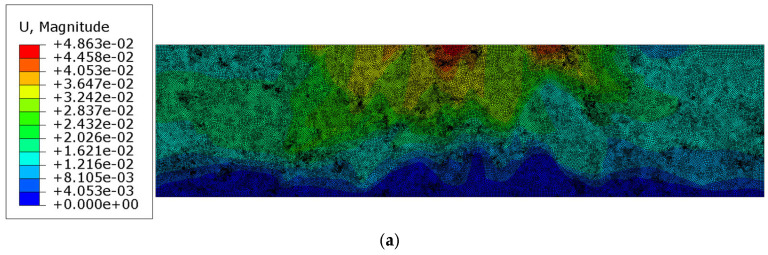

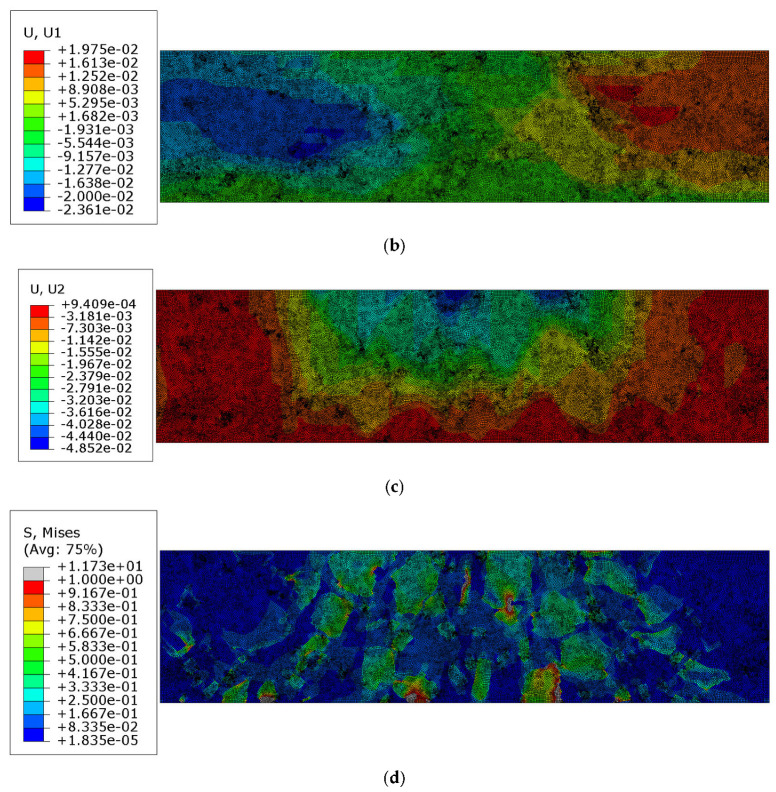
Results of Test 2: (**a**) displacement magnitude [mm]; (**b**) horizontal displacement component $u_{x}$ [mm]; (**c**) vertical displacement component $u_{y}$ [mm]; (**d**) Mises stress [MPa] in a truncated range.

In the next test, the mastic was assumed to be a viscoelastic material with the parameters provided in Table 1, while the inclusions retained the same properties as in the previous test. The analysis was conducted over a period of 150 s. The results obtained at the final time step are presented in Figure 7a–d, corresponding to the reference solution shown in Figure 6a–d.

**Figure 8:**
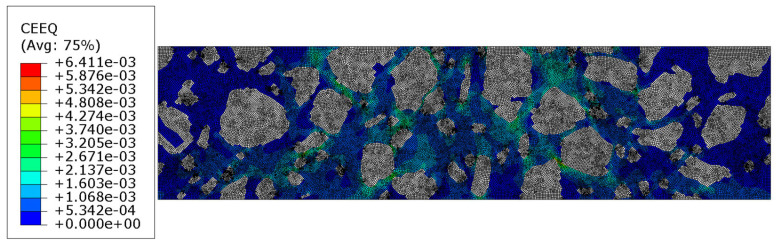
Test 2—equivalent creep strain at the final time step [-].

Additionally, the equivalent creep strain is shown in Figure 8.

### 3.4. Test 3—AC Linear Viscoelastic Analysis with Contact

In the last test, an advanced model was implemented by enhancing the previous test with contact effects at the boundary of each inclusion. This modification allowed us to observe the material’s behavior under the applied load and identify potential areas of delamination. In ABAQUS/Standard 2023, hard contact was applied in the normal direction, while a friction coefficient of 0.3 with the penalty method was used for the tangential direction. This friction coefficient falls within the typical temperature-dependent range of 0.2 to 0.6. The value was carefully selected to place the engineering calculations on the safe side, ensuring conservative and reliable results in the analysis. The finite element model consists of 63,944 nodes and 64,851 plane strain elements, including linear quadrilateral and linear triangular elements. The mesh was optimized and refined to minimize its impact on the simulation outcomes, ensuring accurate and dependable numerical results.

**Figure 9:**
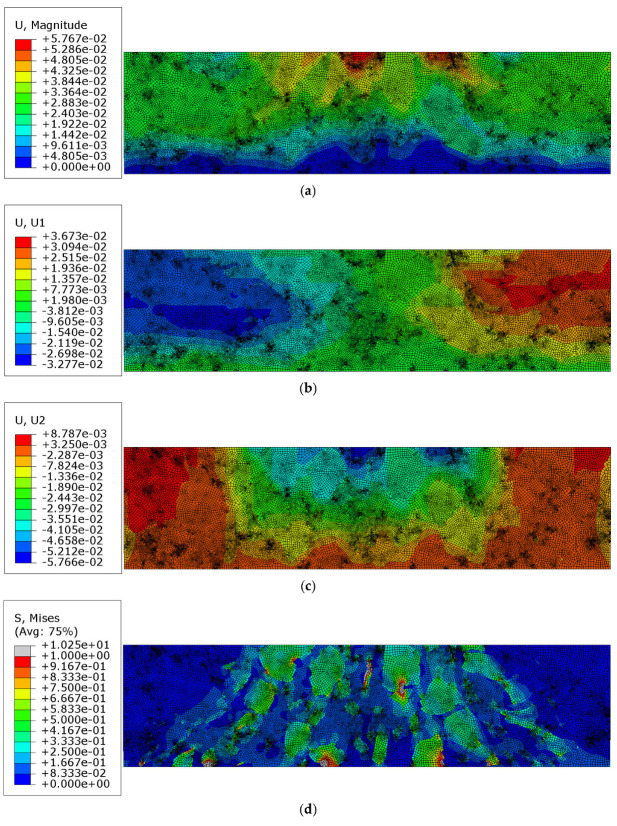
Results of Test 3: (**a**) displacement magnitude [mm]; (**b**) horizontal displacement component $u_{x}$ [mm]; (**c**) vertical displacement component $u_{y}$ [mm]; (**d**) Mises stress [MPa] in a truncated range.

**Figure 10:**
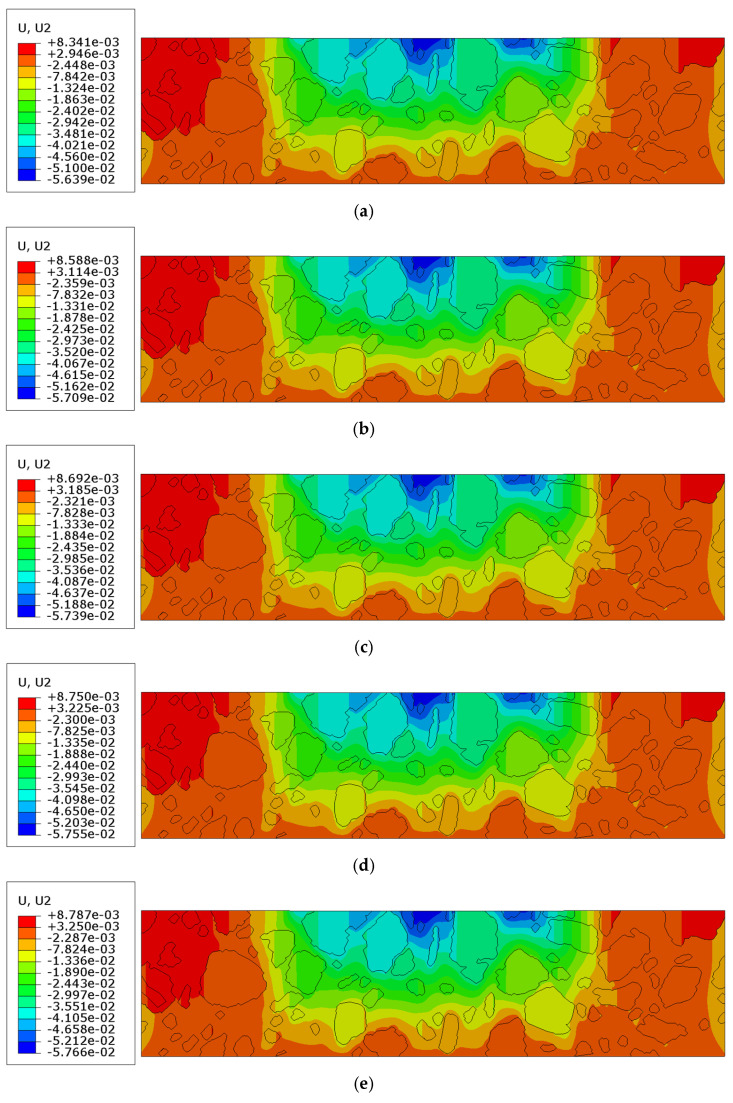
Results of Test 3 (without finite element mesh presentation): vertical displacement component $u_{y}$ [mm] (**a**) for time t = 30 s; (**b**) for t = 60 s; (**c**) for t = 90 s; (**d**) for t = 120 s; (**e**) for t = 150 s.

The analysis was conducted over a period of 150 s. The results obtained at the final time step are presented in Figure 9a–d. They correspond with similar distributions presented for Tests 1 and 2. In Figure 10a–e, the results for the key displacement (the direction of rut formation) in the vertical direction are shown.

**Figure 11:**
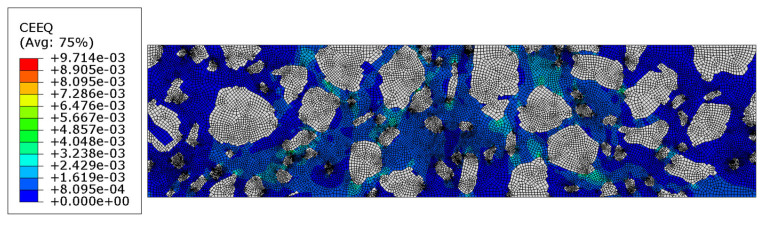
Test 3—equivalent creep strain at the final time step [-].

The equivalent creep strain is shown in Figure 11. It corresponds with a similar distribution shown in Figure 8 for Test 2.

**Figure 12:**
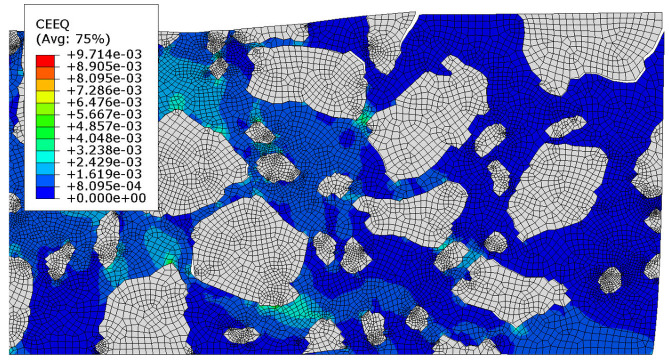
Test 3—equivalent creep strain at the final time step [-]; deformation presented with a scale factor equal to 50.

The zoomed-in distribution of the equivalent creep strain is shown in Figure 12. Therein, a deformation scale factor equal to 50 is used to visualize the occurrence of debonding phenomena in this test.

**Figure 13:**
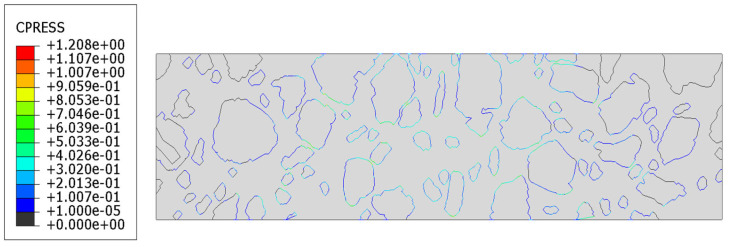
Test 3—contact pressure distribution [MPa].

**Figure 14:**
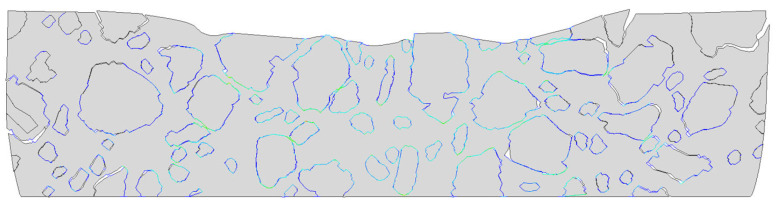
Test 3—contact pressure distribution presented with a scale factor equal to 150.

The contact pressure distribution, i.e., the normal stress transmitted between two surfaces in contact, is shown in Figure 13. It is a critical output variable when analyzing interactions between different materials or components, especially in composite structures where debonding or delamination may occur. A positive value indicates compressive normal stress, meaning the two surfaces are in contact and one is pressing against the other. When the value equals zero, there is no contact—the surfaces are separated, which may be a sign of debonding or loss of cohesion. To better visualize this effect, the results within the domain have been scaled 150 times, as shown in Figure 14.

## 4. Discussion

The results presented in Section 3.1 underscore the importance of accounting for viscous effects in the numerical modeling of asphalt concrete (AC). In this particular test, the elastic response (strain) was approximately equal to only 29% of the viscoelastic response at the final time instance (180 s). The pure linear elastic material model used for the mastic was sufficient only in a very limited range of applications. Practically, the instantaneous response (less than 1 s) can only be reflected by the elastic model. This observation would be even more pronounced if temperature fluctuations were considered in the analysis. In this study, we restricted our analysis by assuming a constant temperature.

Consequently, we used the results presented in Section 3.2 as the reference solution. The specimen microstructure was reconstructed through image processing, utilizing the high-quality image shown in Figure 4 as input. The output geometry, along with the imposed boundary conditions, is illustrated in Figure 5. This geometry was transferred to ABAQUS/Standard 2023 in vector graphics format using our custom Python 3.12 script. The same microstructure was used for Tests 1–3.

The results presented in Section 3.2 were obtained using the assumption of the perfect bonding between the mastic and aggregate particles, with linear elastic behavior assumed for both phases. These results are shown in Figure 6a–d. A lack of symmetry can be observed due to the nonperiodic heterogeneous microstructure of the AC specimen. The minimum dominant (vertical) displacement component value equaled −1.999 × 10^−2^ mm. The extreme horizontal displacement component values yielded +4.090 × 10^−3^ mm/−3.961 × 10^−3^ mm. The maximum reduced von Mises stress for this test was 4.306 MPa.

In Section 3.3, the results of the more complex analysis are presented (Figure 7a–d and Figure 8). Namely, the aggregate particles were modeled as linear elastic, while the mastic was modeled as a viscoelastic material using the Prony series representation with parameters from [36]. Perfect bonding between the constituents was assumed in this example as well. It is clear that the displacement distribution differed slightly from that obtained in Test 1, reflecting the delayed viscoelastic response of the mastic. The analysis period was equal to 150 s in Test 2. Thus, the quantities observed at the final time step were used for the comparison. The minimum vertical displacement component was equal to −4.852 × 10^−2^ mm. The extreme horizontal displacement component values yielded +1.975 × 10^−2^ mm/−2.361 × 10^−2^ mm and the maximum Mises stress was equal to 11.730 MPa at the final analysis step. Additionally, the maximum value of the equivalent creep strain was equal to 6.411 × 10^−3^.

In Section 3.4, the results of the most advanced problem of this study are presented. The model presented in Section 3.3 was enhanced to include the analysis of contact between the mastic and aggregate particles. This modification resulted in further changes to the displacement distribution (Figure 9a–c) compared to the previous tests. It was due to the possible debonding that may occur in the specimen (see Figure 12 and Figure 14) in response to the extreme load subjected. The final values obtained were used for comparison. The minimum vertical displacement component was −5.766 × 10^−2^ mm. The extreme horizontal displacement component values were equal to +3.673 × 10^−2^ mm/−3.277 × 10^−2^ mm and the maximum Mises stress was equal to 10.250 MPa at the final analysis step. The maximum value of the equivalent creep strain was equal to 9.714 × 10^−3^.

From the engineering point of view, the critical values in maintenance systems are associated with vertical displacements. They correspond with the rut depth, which is the basic parameter used for the evaluation of the pavement structure performance. Therefore, for brevity, the vertical displacement components were used for the comparison.

Using the elastic solution as a reference, one can observe the following:

- In Test 2, with the viscoelastic model for the mastic, the minimum vertical displacement increased by 142.72%;
- In Test 3, with the viscoelastic model for the mastic and contact analysis included, the minimum vertical displacement increased by 188.44%.

Additionally, the increase in equivalent creep strain can be observed, amounting to 51.53%, with the Test 2 result as a reference.

It should be considered that one important simplification was made in the numerical tests. Namely, the analysis was carried out in a 2D space (assuming plain strain conditions). It is obvious that aggregate particles are irregularly shaped and 3D microstructure geometry cannot be generated by a simple extrusion operation. For a more realistic representation of the microstructure, its full tridimensional recognition is necessary. High-quality image processing would have to be extended to the processing of XRCT scans or other techniques. It is one of our future research plans to extend the whole framework addressing this issue. Nevertheless, our previous numerical tests [22] confirm the applicability of 2D microstructure to the reliable modeling of AC specimens.

## 5. Conclusions

The concluding remarks of this study are as follows:

- Linear elastic analysis of asphalt concrete is practically insufficient; the viscoelastic behavior of the mastic should be accounted for in the reliable numerical analysis;
- Future enhancements should consider possible debonding between the aggregate particles and mastic, facilitating a more accurate model of asphalt concrete failure;
- Digital reconstruction of asphalt concrete microstructure enables the virtual analysis of realistic specimens.

Further development of the analysis will also require methods for modeling crack propagation within the mastic, which is beyond the scope of the present paper.

While this study focused on the initiation of cracks along the aggregate particles, our future research will aim to integrate these two aspects, proposing a comprehensive framework for the reliable modeling of asphalt concrete accounting for its internal heterogeneous microstructure. Extension of the framework to the 3D microstructure recognition based on XRCT scan processing [37,38,39] and crack propagation modeling within the mastic is the consecutive necessary model enhancement. It can be useful for modeling other phenomena that stem from debonding, e.g., blistering [40].

## Figures and Tables

**Table 1:** Prony series parameters $g_{i}$ and $\tau_{i}$ for the mastic [36].

$g_{i}$ [-]	$\tau_{i}$ [s]
0.1621	0.001
0.267	0.0036
0.144	0.0479
0.099	0.1741
0.064	0.6325
0.0375	2.2974
0.0202	8.3453
0.0097	30.3443
0.0042	110.1169

## Data Availability

The original contributions presented in this study are included in the article. Further inquiries can be directed to the corresponding author.

## Abbreviations

The following abbreviations are used in this manuscript:

AC	asphalt concrete
BVP	boundary value problem
FEA	finite element analysis
FEM	finite element method
ITZ	interfacial transition zone
MsFEM	multiscale finite element method
RVE	representative volume element
SCB	semi-circular bending
XRCT	X-ray computed tomography
